# Intraventricular tuberculosis abscess in an immunocompromised patient: clinical vignette

**DOI:** 10.2478/abm-2021-0036

**Published:** 2021-12-30

**Authors:** Mohamad Hanafiah, Shahizon A Mohamed Mukhari, Aida M Mustapha, Nazimah Ab Mumin

**Affiliations:** Department of Radiology, Sunway Medical Centre, Jalan Lagoon Selatan, Bandar Sunway, 47500 Petaling Jaya, Selangor, Malaysia; Department of Radiology, Hospital Canselor Tuanku Muhriz, Universiti Kebangsaan Malaysia, Jalan Yaacob Latif, Bandar Tun Razak, 56000 Cheras, Kuala Lumpur, Malaysia; Department of Radiology, Hospital Shah Alam, Persiaran Kayangan 7, 40000 Shah Alam, Selangor, Malaysia; Department of Radiology, Faculty of Medicine, University Teknologi Mara, 40450 Shah Alam, Selangor, Malaysia

**Keywords:** abscess, diagnostic imaging, neuroradiology, tuberculosis

## Abstract

Tuberculosis is caused by *Mycobacterium tuberculosis*. Tuberculosis of the central nervous system is common and manifestations include meningeal and intraparenchymal diseases. However, intraventricular tuberculous abscess is a rare manifestation of intracranial tuberculous infection. We present a case of an immunocompromised female patient with high-grade fever and signs of meningism. The computed tomography and magnetic resonance imaging (MRI) of the brain showed hydrocephalus with rim-enhancing lesion in the right lateral ventricle. The MRI demonstrated a hypointense signal on T1-weighted imaging, hyperintense signal on T2-weighted imaging, and mild restricted diffusion in diffusion-weighted imaging. She underwent emergency external ventricular drainage and frank pus was drained. Diagnosis of tuberculosis was made via polymerase chain reaction analysis and culture. Understanding the intracranial manifestation of neurotuberculosis is imperative to arrive at the diagnosis correctly and ensure prompt treatment.

Tuberculosis is caused by *Mycobacterium tuberculosis*. Immunocompromised status in human immunodeficiency viral infection and low socioeconomic status are predisposing factors. Tuberculosis of the central nervous system is common and manifestations include meningeal and intraparenchymal diseases. Intraparenchymal disease usually presents as solitary or multiple tuberculoma [1]. However, tuberculous abscess is an uncommon intraparenchymal manifestation.

Intraventricular involvement by tuberculosis is very rare. We present a case of intraventricular tuberculous abscess in an immunocompromised patient and its imaging findings.

## Case report

A 17-year-old immunocompromised patient presented with fever, vomiting, and headache. She had medical history of B thalassemia with splenectomy at age 7 years, and taking lifelong prophylactic amoxicillin 500 mg twice a day. On admission she was febrile with temperature of 38.8 °C. Her vital signs were stable. There was hyperreflexia of both upper and lower limbs with positive Kernig sign. There was no motor or sensory deficit of either upper or lower limbs. In view of the clinical presentation of meningitis, she received intravenous antibiotics consisting of cefoperazone (Cefobid 2 g intravenously 4 times a day) and vancomycin (1 g intravenously 3 times a day) empirically.

Laboratory blood test results showed low hemoglobin of 7.3 g/dL with leukocytosis (48.4 mmol/L) of predominant neutrophils (75%). The C-reactive protein was raised (12.25 mg/dL). The cerebrospinal fluid analysis showed turbid fluid with increased protein level (3520 mg/L) and low glucose level (1.9 mmol/L, compared with a standard blood glucose level of 7.5 mmol/L). Acid-fast bacilli (AFB) staining of cerebrospinal fluid was negative.

**Figure 1:**
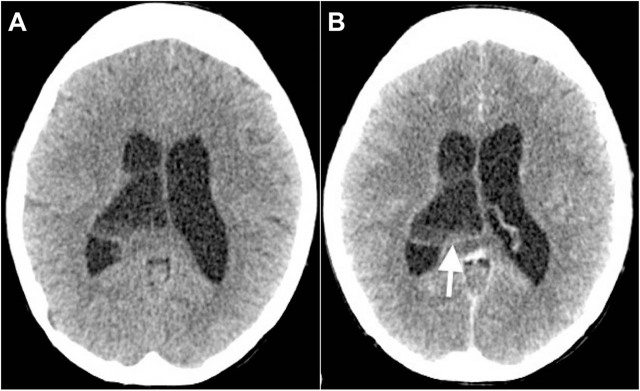
Axial images of (A) plain and (B) contrast computed tomography of the brain show a cystic lesion within the right lateral ventricles with sediments and septations within. There is also a minimal rim enhancement of the wall (arrow) and dilatation of both lateral ventricles consistent with hydrocephalus.

**Figure 2:**
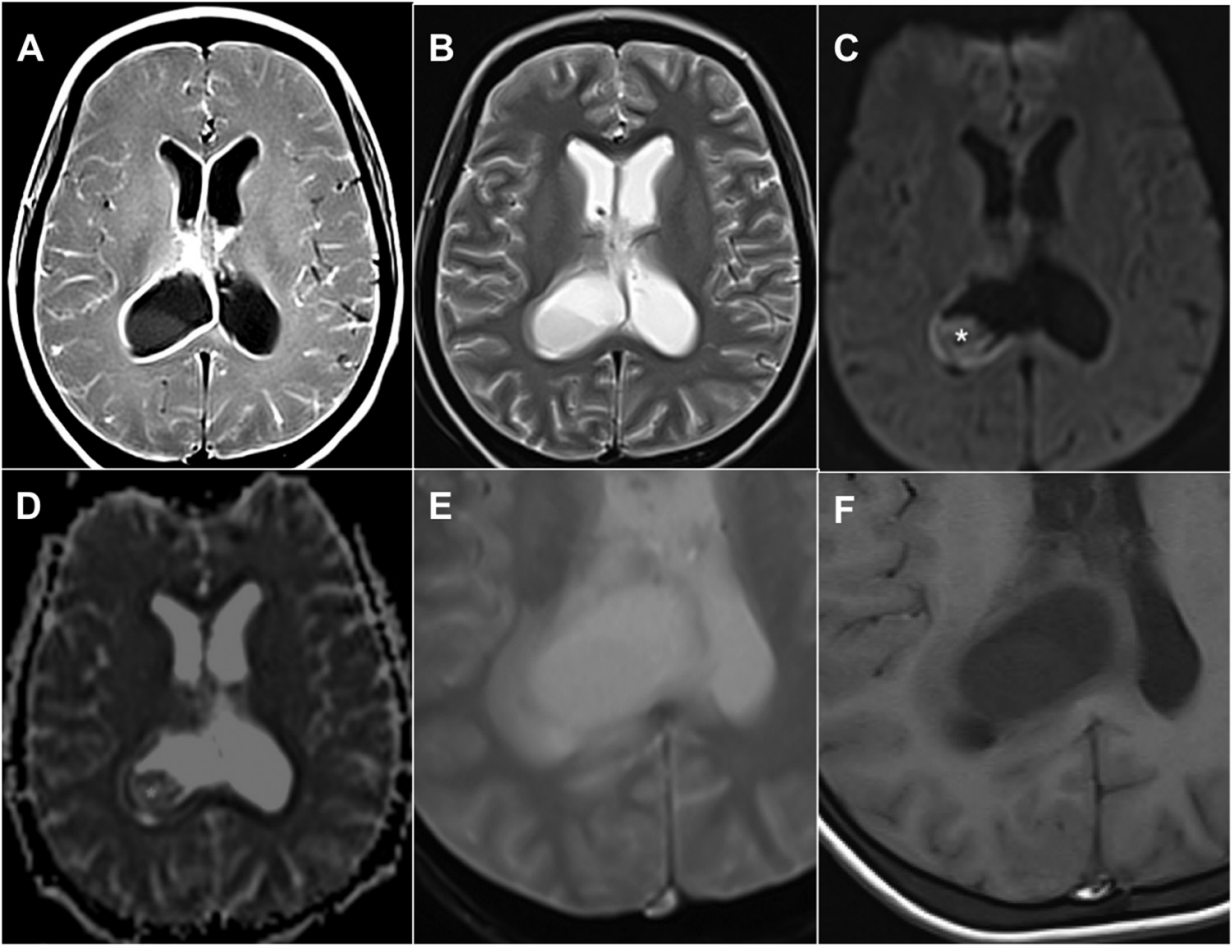
Axial magnetic resonance imaging of the brain shows hydrocephalus and the tuberculous abscess, which is a collection within the posterior horn of the right lateral ventricle. The (A) T1W post contrast image shows rim enhancement of the wall of the lesion, and the ependymal lining of both lateral ventricles and along the cortical gyri. (B) A T2W image shows hyperintense signal of the core of the lesion with an incomplete hypointense rim of the wall. (C) Diffusion-weighted image and the corresponding (D) apparent diffusion coefficient map demonstrate mild diffusion restriction (*) at the inferior aspect of the lesion. Close-up of axial images on (E) T2*-weighted gradient-echo and (F) T1W sequences. No evidence of a hypointense rim is seen in the gradient-echo image. T1W, T1-weighted; T2W, T2-weighted.

Contrast enhanced computed tomography of the brain revealed a cystic lesion, measuring 2.3 cm at the atrium of right lateral ventricle with minimal, thin, and incomplete rim enhancement and obstructive hydrocephalus. There is central nonenhancement of the lesion (**Figure 1**). Magnetic resonance imaging (MRI) of the brain showed that the incomplete and thin rim-enhancing lesion contains fluid-sediment layering within (**Figure 2**). The lesion demonstrated low signal intensity on T1-weighted (T1W) imaging and high signal on T2-weighted (T2W) imaging. The wall of the lesion was isointense on T1W and heterogeneously hypointense on T2W images. Mild restricted diffusion at the inferior part of the lesion was found on diffusion-weighted imaging (**Figure 2**). In addition, the ependymal linings were thickened and enhancing.

External ventricular drainage was performed, and pus drained intraoperatively. The AFB test was negative. However, the PCR analysis and culture results were positive for *M. tuberculosis*. No other source of infection found. Unfortunately, images of the pus drained as evidence were not available for inclusion in this case report. Antituberculous treatment was initiated. She had several episodes of blocked external ventricular drain and reaccumulation of collections requiring several revisions, including the use of endoscopic stereotactic drainage, which revealed an encapsulated thick walled multiloculated collection of frank pus consistent with abscess collection. Histopathology of the capsule showed vascular granulation tissue, predominantly polymorphonuclear leucocytes. During admission, she was started on 2 months of ethambutol (675 mg once a day [OD]), isoniazid (250 mg OD), rifampicin (450 mg OD), and pyrazinamide (1200 mg OD). Corticosteroid was administered intravenously in a titrating dosage of 16 mg/day in the first week to 4 mg/day in the fourth week, then decreasing by 1 mg per week. She gradually improved clinically and was discharged home with 10 months of isoniazid 75 mg and rifampicin 150 mg, and follow-up treatment as an outpatient with the neurology and infectious disease clinics. The consent of the patient (now older than 18 years and also at the time of consent) has been obtained for publication of this case report and any accompanying images.

## Discussion

Despite advancement in medicine, tuberculosis remains a significant infectious disease with approximately 10.4 million new cases and 1.8 million in mortality worldwide in 2016, especially in developing countries [2, 3]. The risk of tuberculosis infection is directly related to exposure to *M. tuberculosis* with higher risk in immunodeficient patients, as they are particularly vulnerable to progression from latent to active disease [4]. Intracranial manifestations of tuberculosis include hydrocephalus, meningeal inflammation, tuberculoma, tuber-culous abscess, cerebritis, vasculitis, cranial neuropathies, and ventriculitis [1]. Intraventricular tuberculous abscess, as presented in the current case, although not so rare in an immunocompromised patients, is still not a common occurrence in tuberculous intracranial infection.

Tuberculous infection of the central nervous system may mimic other inflammatory or neoplastic conditions. The clinical symptoms vary according to the location of infection. A patient with neurological tuberculosis commonly presents with headache, vomiting, convulsions, focal neurological deficit, or visual disturbance. Fever is present only in <10%–20% of presentations [2]. Another conundrum in neurological tuberculosis is that from the clinical diagnosis, cerebrospinal fluid investigation is often normal, and surgical biopsy is an invasive procedure. Clinicians are usually faced with a dilemma in diagnosing intracranial tuberculous infection promptly and confidently. MRI with gadolinium contrast application is a method of choice for initial investigation.

Tuberculomas of the brain are characterized by typical granulomatous reaction consisting of epithelioid cells and giant cells mixed with predominantly lymphocytes [5]. The tuberculomas can be noncaseating, caseating with solid center, or caseating with a liquid center. By contrast, tuber-culous abscess is characterized by an encapsulated collection of pus containing viable bacilli, and the inflammatory reaction in the abscess wall is predominantly vascular granulation tissue containing acute and chronic inflammatory cells [5]. The central multinucleate giant cells surrounded by epithelioid granulomatous reactions that characterize tuberculomas are absent in tuberculous abscess. Any liquefaction within a tuberculoma contains clear or straw-colored fluid, as distinct from pus, which is seen in the tuberculous abscess [5]. The diagnosis of a true tuberculous abscess is usually made using Whitener's criteria, which include macroscopic evidence of cavity formation with central pus, inflammatory reaction in an abscess wall composed primarily of vascular granulation tissue, and proof of tuberculous origin by either culture or acid-fast stain in the pus or abscess wall [6]. The origin of an intraventricular tuberculosis abscess is not clear. The hematogenous spread of infection through the choroid plexus or subependymal region with subsequent spread into the ventricles has been proposed [7]. Once the mycobacterial tuber-cle evokes secondary reaction, it leads to formation of a thick capsule, which may result in a central caseation, liquefaction, and abscess formation. Intracranial tuberculosis abscess formation is more commonly seen in immunocompromised patients as in our case, owing to splenectomy.

The identification of an intracranial lesion can be established by cross-sectional imaging. Although not specific, both computed tomography and MRI are sensitive for detecting tuberculoma or tuberculous abscess. MRI offers higher sensitivity and specificity than computed tomography. On MRI, intracranial caseating solid tuberculoma usually demonstrates central low or isointense signals on T2W images, whereas the noncaseating tuberculoma, liquefied caseating tuberculoma and tuberculous abscess normally reveal T2W central hyper-intensity [8]. The liquefied caseating tuberculoma and tuber-culous abscess often demonstrate hypointense rim on T2W and rim enhancement on postcontrast T1W scans [8, 9, 10]. This appearance is like that for a pyogenic abscess [9]. Additionally, the liquefied tuberculoma or tuberculous abscess often shows restricted diffusion on diffusion-weighted imaging, again like a pyogenic abscess [11] Expectedly, the intraventricular tuberculous abscess as in our case showed hypointensity on T1W, hyperintensity on T2W and rim enhancement. The center of the abscess demonstrated mild restriction diffusion in diffusion-weighted imaging. A previous case report describes a nonenhancing lesion, which was hyperintense on T1W, causing obstructive hydrocephalus [12]. The patient reported in this case underwent ventriculostomy and recovered well with no neurological deficit 6 months after surgery [12]. An intraventricular lesion in a pediatric patient, which was initially diagnosed as a possible brain tumor from the clinical and radiological findings, was revealed as a tuberculoma on surgical excision [13].

## Conclusion

Tuberculosis of the central nervous system poses challenges for both diagnosis and management aspects. Intraventricular tuberculous abscess is a rare manifestation of intracranial tuberculous infection. The appearance of intraventricular tuberculous abscess on conventional MRI is like that for other intraparenchymal tuberculous or pyogenic abscesses. Understanding the typical and atypical intracranial manifestation of neurotuberculosis is imperative to arrive at the diagnosis correctly and ensure prompt treatment given.
